# Competing risks survival analysis applied to data from the Australian Orthopaedic Association National Joint Replacement Registry

**DOI:** 10.3109/17453674.2010.524594

**Published:** 2010-10-08

**Authors:** Marianne H Gillam, Philip Ryan, Stephen E Graves, Lisa N Miller, Richard N de Steiger, Amy Salter

**Affiliations:** ^1^School of Population Health and Clinical Practice; ^2^Data Management and Analysis Centre, Discipline of Public Health, University of Adelaide; ^3^Australian Orthopaedic Association National Joint Replacement Registry, Adelaide, Australia

## Abstract

**Background and purpose:**

The Kaplan-Meier (KM) method is often used in the analysis of arthroplasty registry data to estimate the probability of revision after a primary procedure. In the presence of a competing risk such as death, KM is known to overestimate the probability of revision. We investigated the degree to which the risk of revision is overestimated in registry data.

**Patients and methods:**

We compared KM estimates of risk of revision with the cumulative incidence function (CIF), which takes account of death as a competing risk. We considered revision by (1) prosthesis type in subjects aged 75–84 years with fractured neck of femur (FNOF), (2) cement use in monoblock prostheses for FNOF, and (3) age group in patients undergoing total hip arthroplasty (THA) for osteoarthritis (OA).

**Results:**

In 5,802 subjects aged 75–84 years with a monoblock prosthesis for FNOF, the estimated risk of revision at 5 years was 6.3% by KM and 4.3% by CIF, a relative difference (RD) of 46%. In 9,821 subjects of all ages receiving an Austin Moore (non-cemented) prosthesis for FNOF, the RD at 5 years was 52% and for 3,116 subjects with a Thompson (cemented) prosthesis, the RD was 79%. In 44,365 subjects with a THA for OA who were less than 70 years old, the RD was just 1.4%; for 47,430 subjects > 70 years of age, the RD was 4.6% at 5 years.

**Interpretation:**

The Kaplan-Meier method substantially overestimated the risk of revision compared to estimates using competing risk methods when the risk of death was high. The bias increased with time as the incidence of the competing risk of death increased. Registries should adopt methods of analysis appropriate to the nature of their data.

Arthroplasty registries typically present results of joint replacement in terms of the Kaplan-Meier (KM) estimates of the survival of the primary prosthesis. The estimates are interpreted as the probability of the prosthesis surviving until a nominated time after implantation. Alternatively, a registry may quote the complement (in probability) of the KM survivorship function. In the Australian Orthopaedic Association National Joint Replacement Registry (AOA NJRR) (AOANJRR 2009), this latter measure of revision is termed the “cumulative per cent revision” (CPR).

A registry follows up patients from the date of the primary procedure until the date of statistical analysis. The observation time of a patient who has undergone a primary procedure but who has not had a revision by the date of analysis is said to be right censored at that date. We do not know when, in the future, that patient may undergo a revision. All we know is that it has not happened yet, and the KM method takes that into account using all the information on that patient up until the date of censoring. Crucially, the KM method assumes that patients whose time is censored will have the same chance of revision at any subsequent time as those whose time is not censored. In a sense, censoring is an inconvenience that prevents us from seeing what may happen in the future.

The problem with the use of the KM method in the analysis of registry data is that deaths are handled in exactly the same way: the patient's follow-up time is right censored at the time of death. However, death differs from censoring in that it does not merely conceal the occurrence of a future revision, it changes the probability of revision occurring. Essentially, under the KM method we are assuming that dead patients will have the same chance of eventually having a revision as those still living.

When a patient is at risk of experiencing multiple events, with each precluding the other events or altering the probability of occurrence of the other events, these events are called competing risks (Gooley et al. 1999). Death changes the probability of a patient's prosthesis being revised and is said to be a competing risk for revision, the event of interest. Similarly, revision is a competing risk to death as it precludes the occurrence of death as a first event.

The above mentioned problem with the KM method has sometimes been approached by pretending that the competing event, in this case death, can be removed and by assuming that the revision rate is unaffected by this. However, since it is impossible to know from the data at hand how removing one outcome would affect the outcome of the other event(s), this is purely speculative (Prentice et al. 1978, Andersen et al. 2002). Furthermore, since there is a negative correlation between the likelihood of undergoing a revision and death, the 2 events are not independent. The implication of the violation of the KM assumptions is that the KM estimates in the presence of competing risks do not have a meaningful probability interpretation (Andersen et al. 2002); that is, the KM estimate of revision is not a valid estimate of the probability of revision assuming that the patient does not die.

Kalbfleisch and Prentice (1980) have developed a method for estimating the probability of revision in the competing risks situation, based on a measure called the cumulative incidence function (CIF). The CIF for revision at any time depends on both the number of patients who have been revised and the number of patients who have not experienced any event (death or revision) by that time. Hence, when the CIF is used to estimate the probability of revision, the probability of death is taken into account. Patients who have neither died nor been revised by the date of analysis are treated as right censored, just as with the KM method.

In the presence of a competing risk such as death, the standard KM method will always overestimate the true revision rate (Biau et al. 2007, Putter et al. 2007). If the death rate is low, then the bias in estimating the risk of revision using KM is small. But in elderly and frail patients, or in registry data where long-term observation is the goal, the competing risk of death becomes greater and the magnitude of the KM overestimate of revision will become more substantial.

In this study, we applied methods of competing risk to data from large cohorts of patients in the AOA National Joint Replacement Registry and contrasted the results with those obtained from the standard Kaplan-Meier method.

## Materials and methods

### Material

Data for this study were obtained from the Australian Orthopaedic Association National Joint Replacement Registry (AOA NJRR). The registry was established and began data collection in 1999, and achieved full national coverage in 2002. The AOA NJRR now has data on 550,000 procedures. Almost 100% of hip and knee procedures performed in Australia are captured. Mortality data in the AOA NJRR are obtained from the National Death Index, a database maintained by the Australian Institute of Health and Welfare.

Our data consisted of records of patients who received partial or total arthroplasty for fractured neck of femur (FNOF) and of patients who received total hip arthroplasty (THA) for osteoarthritis (OA) in the 7-year period from January 1, 2002 to December 31, 2008. We chose 3 areas of interest for our analysis. In the FNOF group, we examined results from 2 subsets of data. In the first, we compared results from 4 main types of prostheses (monoblock, unipolar modular, bipolar, and THA) in the age group 75–84 years; in the second, we compared results from 2 types of monoblock prostheses (cementless Austin Moore and cemented Thompson) in all ages. For our third set of analyses, based on patients with osteoarthritis who underwent THA, we compared results from patients younger than 70 years with results from patients who were 70 years or older. For all analyses, we excluded patients who had bilateral procedures. Revisions are reoperations of primary hip replacements and involve removal and/or replacement of one or more component used in the primary procedure.

### Statistics

The outcome of interest was “time to first revision”, being the time interval between the date of insertion of the primary prosthesis and the date of revision. We used standard Kaplan-Meier survival analysis to calculate the cumulative per cent of primary procedures revised (CPR) (the Kaplan-Meier (KM) estimates). To take account of the competing risk of death, we calculated the cumulative incidence function (CIF). Confidence intervals for the CPR were based on a method developed by Kalbfleisch and Prentice (1980) and the confidence intervals for the CIF were based on the method of Aalen (1978).

In the non-competing risk paradigm, we used the log-rank test to test for differences in CPR, and in the competing risk paradigm we used Gray's test (1988) to compare the CIF between groups.

In order to give an indication of the magnitude of the overestimation (that is, the bias) of the KM estimate, we calculated the difference of the KM and CIF estimates, and the per cent relative difference (RD):


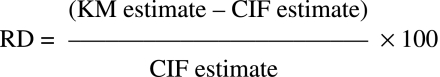


The estimates of CPR, CIF, difference, and RD are displayed in graphs and tables. As recommended when dealing with competing risks, we show plots of mortality and also of revisions (Dignam and Kocherginsky 2008). Plots were curtailed when the number at risk became small (Tables 2, 3, and 4), following the conventions adopted in the Australian Registry's Annual Report (AOANJRR 2009) (p3) and those recommended by Pocock et al. (2002). However, for the test statistics we used all available data.

## Results

The distributions of outcomes (censoring, revision, and death) for patients in the 3 groups of interest are presented in Table 1. Note that the percentage revised shown in this table is the simple raw percentage for descriptive purposes, and not the KM or CIF estimate.

**Table 1.** Distribution of outcomes for the three study groups

	Censored **^a^**	Revised **^b^**	Deceased	Total
FNOF, prosthesis types (75–84 years of age)				
Monoblock	2,348 (40%)	225 (4%)	3,229 (56%)	5,802
Unipolar	2,521 (70%)	98 (3%)	990 (27%)	3,609
Bipolar	1,921 (62%)	73 (2%)	1,114 (36%)	3,108
THA	1,373 (76%)	64 (4%)	368 (20%)	1,805
Total	8,163 (57%)	460 (3%)	5,701 (40%)	14,324
FNOF, monoblock (all ages)				
Austin Moore	3,310 (34%)	369 (4%)	6,142 (63%)	9,821
Thompson	1,283 (41%)	75 (2%)	1,758 (56%)	3,116
Total	4,593 (36%)	444 (3%)	7,901 (61%)	12,937
OA, THA				
Age < 70 years	42,136 (95%)	1,149 (3%)	1,080 (2%)	44,365
Age ≥ 70 years	41,623 (88%)	1,189 (3%)	4,618 (10%)	47,430
Total	83,759 (91%)	2,338 (3%)	5,698 (6%)	91,795

**^a^** right censored due to closure of database for analysis.

**^b^** simple raw proportion, not allowing for censoring.

FNOF: fractured neck of femur; OA: osteoarthritis; THA: total hip arthroplasty.

**Figure 1.**
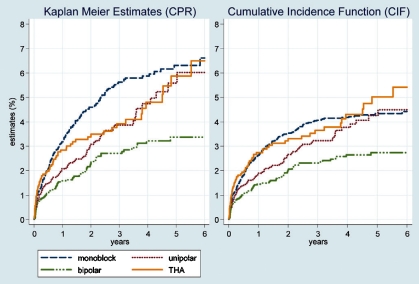
Estimates of revision by type of prosthesis in patients with FNOF who were aged 75–84 years.

**Figure 2.**
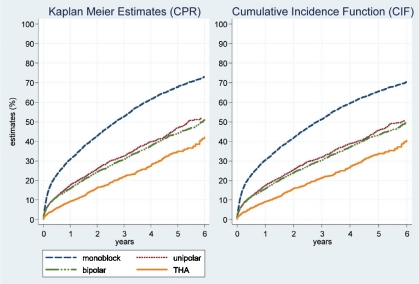
Estimates of death by type of prosthesis in patients with FNOF who were aged 75–84 years.

Figure 1 shows the KM and CIF estimates for revision for the 4 types of prosthesis in patients with FNOF in the 75–84-year age group. The KM estimates were higher than the CIF estimates at each time point for each type of prosthesis (Table 2). The KM and CIF estimates for death are shown in Figure 2. Patients with monoblock prostheses had the highest probability of death. The KM and CIF estimates of death were almost identical but in all cases the KM estimate exceeded the CIF.

**Table 2.** Per cent estimates (with 95% confidence interval) of revision in patients aged 75–84 years with FNOF

	1 year	2 years	3 years	4 years	5 years	6 years
Monoblock						
At risk	3,512	2,521	1,759	1,108	599	266
KM **^a^**	3.2 (2.7–3.8)	4.6 (4.0–5.3)	5.6 (4.9–6.5)	5.9 (5.1–6.8)	6.3 (5.5–7.3)	6.6 (5.6–7.8)
CIF **^b^**	2.6 (2.2–3.1)	3.5 (3.1–4.1)	4.1 (3.6–4.6)	4.2 (3.7–4.8)	4.3 (3.8–4.9)	4.4 (3.9–5.0)
Diff. **^c^**	0.6	1.1	1.5	1.7	2.0	2.2
RD **^d^**	21%	30%	38%	40%	46%	50%
Unipolar						
At risk	2,180	1,376	805	439	223	71
KM	2.1 (1.6–2.7)	3.1 (2.5–3.9)	3.9 (3.1–4.8)	4.8 (3.7–6.0)	5.6 (4.3–7.3)	6.0 (4.5–8.0)
CIF	1.9 (1.5–2.4)	2.7 (2.1–3.3)	3.2 (2.6–4.0)	3.8 (3.0–4.7)	4.3 (3.3–5.3)	4.5 (3.5–5.7)
Diff.	0.2	0.4	0.6	1.0	1.3	1.5
RD	10%	15%	20%	26%	31%	34%
Bipolar						
At risk	2,351	1,868	1,398	951	553	245
KM	1.6 (1.2–2.1)	2.3 (1.7–2.9)	2.7 (2.1–3.5)	3.2 (2.5–4.1)	3.4 (2.6–4.3)	3.4 (2.6–4.3)
CIF	1.5 (1.1–1.9)	2.0 (1.5–2.6)	2.3 (1.8–2.9)	2.7 (2.1–3.3)	2.7 (2.2–3.4)	2.7 (2.2–3.4)
Diff.	0.1	0.3	0.4	0.6	0.6	0.6
RD	9%	14%	17%	21%	23%	23%
THA						
At risk	1,238	873	598	379	215	93
KM	2.8 (2.1–3.8)	3.5 (2.7–4.6)	3.9 (3.0–5.1)	4.8 (3.6–6.4)	5.8 (4.3–8.0)	6.5 (4.7–9.1)
CIF	2.7 (2.0–3.6)	3.3 (2.5–4.3)	3.7 (2.8–4.7)	4.3 (3.3–5.6)	5.0 (3.7–6.6)	5.4 (4.0–7.2)
Diff.	0.1	0.2	0.3	0.5	0.8	1.1
RD	4%	6%	8%	12%	17%	20%

**^a^** Kaplan-Meier estimate of cumulative per cent revised.

**^b^** Cumulative incidence function.

**^c^** Difference (bias of the KM estimate).

**^d^** Relative difference (bias of KM estimate relative to the CIF).

**Figure 3.**
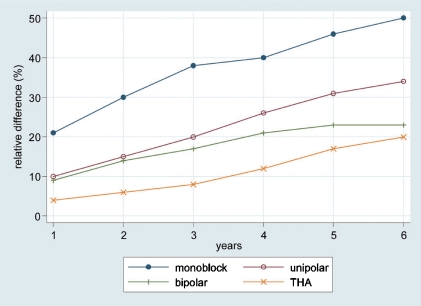
Relative overestimation of KM estimates compared to CIF estimates by years after primary procedure and type of prosthesis; patients were aged 75–84 years with FNOF.

The difference in the KM and CIF estimates of revision was most pronounced for the monoblock prostheses, the group with the highest mortality, and least for THA, the group with the lowest mortality (Table 2 and Figure 2). In all groups, the bias in the KM estimation increased with time as the competing risk of death increased (Figure 3).

The global tests (log-rank test for KM estimates and Gray's test for CIF estimates) comparing revision across all four types of prostheses were statistically significant (p < 0.001 and p = 0.001, respectively). The log-rank test and Gray's test gave the same results for tests between specific pairs of prostheses in all but one of the comparisons. The log-rank test for the difference in revision rate between monoblock and unipolar prostheses was significant (p = 0.006), but the Gray's test for difference between the CIFs was not (p = 0.1).

**Figure 4.**
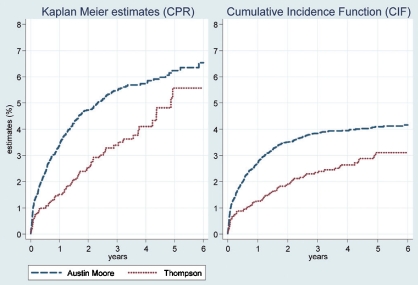
Estimates of revision by type of prosthesis (cementless Austin Moore vs. cemented Thompson) in patients with FNOF.

**Figure 5.**
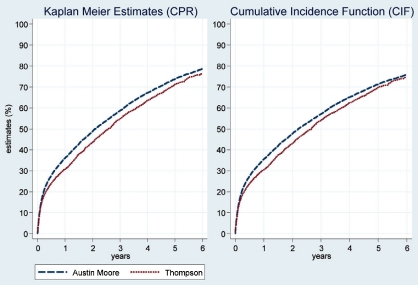
Estimates of death by type of prosthesis (cementless Austin Moore vs. cemented Thompson) in patients with FNOF.

The second group of interest was patients who received either cementless Austin Moore prostheses or cemented Thompson prostheses after FNOF. The KM and CIF estimates in Figure 4 again reveal that the KM method overestimated the risk of revision compared to the CIF in this population. The estimates for revision for each year were highest for those patients who received Austin Moore cementless prostheses (Table 3), but after 5 years the KM method overestimated the risk of revision by more than 2% for both types of prosthesis, and the relative biases were substantial. As can be seen in Figure 5, patients with Austin Moore prostheses had the highest mortality in the study period. Both the log-rank test and Gray's test were significant for difference in revision (p < 0.001) and for difference in death (p < 0.001).

**Table 3.** Per cent estimates (with 95% confidence interval) of revision in patients with FNOF who received Austin-Moore or Thompson prostheses

	1 year	2 years	3 years	4 years	5 years	6 years
Austin Moore stem						
At risk	5,514	3,893	2,756	1,740	963	403
KM **^a^**	3.4 (3.0–3.8)	4.7 (4.2–5.3)	5.5 (4.9–6.1)	5.7 (5.2–6.4)	6.2 (5.6–7.0)	6.5 (5.8–7.4)
CIF **^b^**	2.7 (2.4–3.1)	3.5 (3.1–3.9)	3.8 (3.5–4.3)	3.9 (3.6–4.4)	4.1 (3.7–4.5)	4.2 (3.8–4.6)
Diff. **^c^**	0.7	1.2	1.6	1.8	2.1	2.4
RD **^d^**	25%	35%	42%	45%	52%	57%
Thompson stem						
At risk	1,905	1,334	865	506	230	96
KM	1.5 (1.1–2.1)	2.5 (1.9–3.3)	3.4 (2.6–4.4)	4.1 (3.2–5.3)	5.6 (4.1–7.5)	5.6 (4.1–7.5)
CIF	1.3 (0.9–1.7)	1.9 (1.5–2.5)	2.3 (1.8–3.0)	2.6 (2.1–3.3)	3.1 (2.4–3.9)	3.1 (2.4–3.9)
Diff.	0.3	0.6	1.1	1.5	2.5	2.5
RD	20%	33%	44%	56%	79%	79%

**^a^** Kaplan-Meier estimate of cumulative per cent revised.

**^b^** Cumulative incidence function.

**^c^** Difference (bias of the KM estimate).

**^d^** Relative difference (bias of KM estimate relative to the CIF).

**Figure 6.**
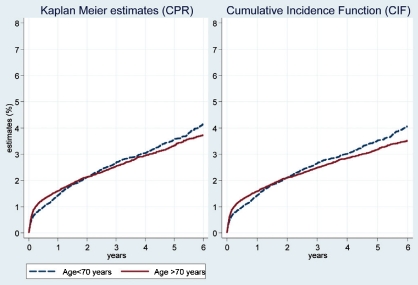
Estimates of revision by age group in patients with OA and THA.

**Figure 7.**
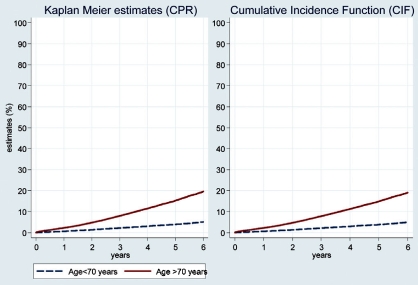
Estimates of death by age group in patients with OA and THA.

Results from the third study group, which consisted of patients who received THA as treatment for OA, are presented in Figures 6 and 7 and in Table 4. The KM estimates in the older age group (70 years and over) increased slightly more with time than the CIF estimates, whereas the KM and CIF estimates in the younger age group (less than 70 years) were very similar. There was no significant difference between the two age groups with respect to revision (Gray's test, p = 0.2), whereas the risk of death was of course higher in the 70-year and older age group (Gray's test, p < 0.001). Overall, the risk of death in this group was substantially lower than in the 2 other study groups (Figures 2 and 5): the competing risk of death was less and so the bias of the KM estimate was less.

**Table 4.** Per cent estimates (with 95% confidence interval) of revision in patients with osteoarthritis who underwent total hip replacement–by age group

	1 year	2 years	3 years	4 years	5 years	6 years
Age < 70 years						
At risk	35,335	27,994	21,454	15,322	9,737	4,714
KM ^**a**^	1.4 (1.3–1.5)	2.1 (2.0–2.3)	2.7 (2.5–2.8)	3.0 (2.9–3.2)	3.6 (3.4–3.8)	4.1 (3.9–4.4)
CIF **^b^**	1.4 (1.3–1.5)	2.1 (2.0–2.3)	2.6 (2.5–2.8)	3.0 (2.8,3.2)	3.5 (3.3–3.7)	4.1 (3.8–4.4)
Diff. **^c^**	0.003	0.01	0.02	0.03	0.05	0.07
RD **^d^**	0.3%	0.5%	0.8%	1.0%	1.4%	1.8%
Age ≥ 70 years						
At risk	38,089	30,254	22,853	16,141	10,059	4,636
KM	1.6 (1.5–1.7)	2.1 (2.0–2.3)	2.5 (2.4–2.7)	2.9 (2.8–3.1)	3.3 (3.1–3.5)	3.7 (3.5–4.0)
CIF	1.6 (1.5–1.7)	2.1 (2.0–2.2)	2.5 (2.3–2.6)	2.8 (2.7–3.0)	3.2 (3.0–3.4)	3.5 (3.3–3.7)
Diff.	0.01	0.03	0.06	0.10	0.15	0.22
RD	0.9%	1.4%	2.3%	3.4%	4.6%	6.2%

**^a^** Kaplan-Meier estimate of cumulative per cent revised.

**^b^** Cumulative incidence function.

**^c^** Difference (bias of the KM estimate).

**^d^** Relative difference (bias of KM estimate relative to the CIF.

## Discussion

We have shown using data from the AOA National Joint Replacement Registry that the KM method overestimated the risk of revision compared to the CIF estimates, and that this was most pronounced when the incidence of the competing risk (death) was high. Furthermore, with time the difference and relative difference between the 2 estimates increased as the incidence of death increased (Figure 3).

In most of the instances where we compared revisions between groups—for example, age groups or prosthesis type—if the KM estimate was higher in one group than in another, so too (in general) would the CIF be. But this was not always the case, and it depended on the distribution of deaths in the groups being compared. Statistical tests for differences in estimates between groups may also yield different results. This is illustrated in the comparison of revisions of monoblock with unipolar prostheses in the 75–84-year age group (Figure 1). The log-rank test for difference in KM estimates was statistically significant whereas the Gray's test for difference in CIF estimates was not. This is because patients with monoblock prostheses had higher mortality than patients with unipolar prostheses (Figure 2) and the CIF estimate depends on both the event of interest and the competing event, whereas the KM estimate depends only on the event of interest. It follows that in the presence of competing risks, it is important to interpret the CIF estimates of the event of interest together with the CIF of the competing event. The CIF estimate may be low for one event because the failure rate for the competing event is high, leading to fewer subjects being left to experience the event of interest. This implies that one has to be cautious when applying results from competing risks analysis on one population to another population with a possible difference in incidence of the competing risks.

Our work has not taken in to account other possible competing risks for the event of interest—revision—but to the extent that they may be present and unaccounted for, our estimates of the KM bias are conservative. Also, we have not considered selection of patients by surgeons on clinical grounds or other characteristics and the biases that may have arisen from that—but neither does the standard KM estimate.

There has been infrequent use of competing risk analysis in orthopedic research. In a study of a real data set and a fictitious data set of hip arthroplasty, Schwarzer et al. (2001) concluded that death should be treated as a competing risk in the analysis, and that the CIF should be used instead of the KM estimate (CPR) to calculate the probability of revision. Biau et al. (2007) discussed the use of the Kaplan-Meier method and the cumulative incidence function to estimate the survival of hip and knee arthroplasty. More recently, Ranstam and Robertsson (2010) from the Swedish Knee Arthroplasty Register have discussed the problem of competing risks as part of a general summary of issues facing statistical analysis of registry data. Using simulated data and a cohort (n=406) of patients undergoing total hip replacement, Fennema and Lubsen (2010) examined the bias of the KM estimate and recommended use of the cumulative incidence in the presence of competing risks.

Competing risk methodology is being increasingly applied to other areas of medical research (Kim et al. 2006, Resche-Rigon et al. 2006, Beyersmann et al. 2007, Wolbers et al. 2009, Evans et al. 2010). One of the limitations in performing competing risks analysis has been the lack of readily available software. This is now changing: Stata has user-written programs for calculating the CIF for competing risks (Coviello and Boggess 2002). The “R” package “cmprsk” contains commands for calculating the CIF and performing Gray's k-sample test. SAS macros also exist for estimating the CIF and performing Gray's test (Moeschberger et al. 2008).

We used only non-parametric methods to estimate the risk of revision in the selected population, as the main goal was to illustrate how the standard KM method overestimates the risk of revision in the presence of a competing risk. Modeling techniques that allow direct comparisons of the cumulative incidence function adjusting for covariates are now also available (Fine and Gray 1999, Klein and Andersen 2005, Zhang and Fine 2008), and we have planned the application of these methods to joint registry data for a later study.

In our data, the differences between the KM and CIF estimates in the 2 FNOF groups were evident after a relatively short time. Scope for further research would entail applying competing risks methodology to joint replacement registry data on patients with lower mortality rates such as in the THA, OA group, but with a much longer follow-up time—where death as a competing risk would thus become more important. Another application of competing risk methods to arthroplasty data is in the analysis of cause-specific revisions, where each cause (infection, dislocation, loosening, etc.) acts as a competing risk to every other cause. If the revision rate due to some of these competing causes of risk is high, the bias of the KM estimate would be considerable. However, in the present study where the revision rate was low, if death were not to be considered a competing risk event, we would expect the difference between the KM and CIF estimates for the different causes of revision to be negligible.

How, then, should clinicians and other readers interpret the results currently published in registry reports, given that the Kaplan-Meier method is used in all national collections? The answer must be “cautiously”. In older patients, in analyses of revisions in frail patients and when a registry has a long follow-up time, caution should be greatest, as overestimation of risk will be highest because of the high incidence of death. Health services planners using estimates of revision from registry reports to project service use and costs should consult the relevant registry and seek statistical advice.

Competing risks present arthroplasty registries with at least 3 related problems. First, the methods of analysis currently being used are known to be inappropriate. Second, the interpretation of KM estimates in the presence of competing risks is difficult. Third, risks of revision may be substantially biased (although in certain circumstances, for many analyses the bias of the KM method will be small and the conclusions will not change). As a result of these problems, registries may be open to criticism from industry, regulatory bodies, and other stakeholders, and this may compromise the undoubted usefulness of the registries.

How should registries respond? They are now aware of the concept of competing risk and the problems of the KM estimate. Indeed, for several years the Swedish Knee Arthroplasty Register has advised readers of their Annual Reports of the bias in published KM estimates (SKAR 2008). However, because of the long and successful histories of many national collections, including the AOA NJRR, and the familiarity of their stakeholders with traditional methods of analysis and presentation, registries have yet to adopt the newer methods. What is needed now is a cooperative effort across all national arthroplasty registries to update their analytical approaches to be in line with current statistical knowledge, coupled with a well-planned and targeted education process for those who make use of the information provided by the registries.
